# Brain volume in chronic ketamine users — relationship to sub-threshold psychotic symptoms and relevance to schizophrenia

**DOI:** 10.1007/s00213-021-05873-0

**Published:** 2021-07-06

**Authors:** Robert A. Chesters, Fiona Pepper, Celia Morgan, Jonathan D. Cooper, Oliver D. Howes, Anthony C. Vernon, James M. Stone

**Affiliations:** 1 Institute of Psychiatry, Psychology and Neuroscience, King’s College London, London, UK; 2 Department of Basic and Clinical Neuroscience, Maurice Wohl Clinical Neuroscience Institute, London, SE5 8AF UK; 3 University of Exeter, Exeter, UK; 4 Departments of Pediatrics, Genetics and Neurology, Medical School, Washington University in St Louis, 660S Euclid Ave, St Louis, MO 63110 USA; 5 South London and Maudsley NHS Trust, London, SE5 8AZ UK; 6 MRC Centre for Neurodevelopmental Disorders, King’s College London, London, SE1 1UL UK; 7 Department of Neuroscience, Brighton and Sussex Medical School, University of Sussex, Falmer, BN1 9RY UK; 8 Sussex Partnership NHS Foundation Trust, Eastbourne, BN21 2UD UK

**Keywords:** Ketamine, Brain volume, MRI, Schizophrenia, Psychosis

## Abstract

**Rationale:**

Ketamine may model aspects of schizophrenia arising through NMDA receptor activity deficits. Although acute ketamine can induce effects resembling both positive and negative psychotic symptoms, chronic use may be a closer model of idiopathic psychosis.

**Objectives:**

We tested the hypotheses that ketamine users had lower brain volumes, as measured using MRI, and greater sub-threshold psychotic symptoms relative to a poly-drug user control group.

**Methods:**

Ketamine users (n = 17) and poly-drug using controls (n = 19) were included in the study. All underwent volumetric MRI imaging and measurement of sub-threshold psychotic symptoms using the Comprehensive Assessment of At-Risk Mental State (CAARMS). Freesurfer was used to analyse differences in regional brain volume, cortical surface area and thickness between ketamine users and controls. The relationship between CAARMS ratings and brain volume was also investigated in ketamine users.

**Results:**

Ketamine users were found to have significantly lower grey matter volumes of the nucleus accumbens, caudate nucleus, cerebellum and total cortex (FDR p < 0.05; Cohen’s d = 0.36–0.75). Within the cortex, ketamine users had significantly lower grey matter volumes within the frontal, temporal and parietal cortices (Cohen’s d 0.7–1.31; FDR p < 0.05). They also had significantly higher sub-threshold psychotic symptoms (p < 0.05). Frequency of ketamine use showed an inverse correlation with cerebellar volume (p < 0.001), but there was no relationship between regional brain volumes and sub-threshold psychotic symptoms.

**Conclusions:**

Chronic ketamine use may cause lower grey matter volumes as well as inducing sub-threshold psychotic symptoms, although these likely arise through distinct mechanisms.

**Supplementary Information:**

The online version contains supplementary material available at 10.1007/s00213-021-05873-0.

## Introduction

There has been considerable interest in the role of N-methyl-D-aspartate (NMDA) receptor dysfunction in schizophrenia. This idea originally arose through the observation that NMDA receptor antagonists such as phencyclidine (PCP) and ketamine induced psychosis-like effects resembling both the positive and negative symptoms of schizophrenia (Javitt 2007; Krystal et al. 1994). Subsequent work highlighted the finding that NMDA receptor antagonists led to neurotoxic changes in rodents, which was suggested to resemble volume reductions seen in patients with schizophrenia and which was blocked by AMPA receptor antagonists, suggesting that these changes were driven, in part, by increased synaptic glutamate release (Olney and Farber 1995). Since then, there has been some evidence that NMDA receptor dysfunction might be present in patients with schizophrenia including post-mortem findings of reduced NMDA receptor mRNA in frontal cortex (Sokolov 1998), temporal cortex (Humphries et al. 1996) and hippocampus (Law and Deakin 2001) and reductions in in vivo hippocampal NMDA receptor binding in unmedicated patients with schizophrenia (Pilowsky et al. 2006).

It has been suggested that ketamine administration might model some of the features of schizophrenia, with the acute effects of ketamine resembling those seen in the prodrome of psychosis (Abi-Saab et al. 1998; Corlett et al. 2007) and exacerbating psychotic symptoms in patients with existing schizophrenia (Lahti et al. 2001; Malhotra et al. 1997). On the other hand, chronic ketamine and PCP users have been reported to have symptoms more closely resembling established schizophrenia, including paranoid delusions, auditory hallucinations and cognitive deficits (Abi-Saab et al. 1998; Javitt 2007; Jentsch and Roth 1999; Krystal et al. 1994; Morgan et al. 2012).

Brain volume reductions in frontal and temporal cortices have been reported in the transition between at risk mental state and first episode psychosis (Chung and Cannon 2015) and further reductions in frontal, parietal and temporal cortices have been reported in first episode and chronic schizophrenia (Vita et al. 2012). If chronic ketamine use is a model for schizophrenia, it might be predicted that, as well as having psychosis-like symptoms, these individuals would show a similar pattern of brain volume loss to patients with schizophrenia. Hence, one key question is whether chronic ketamine use is associated with lower brain volume compared to healthy volunteers. Our current understanding of the effects of ketamine on the brain structure is, however, poor. To the best of our knowledge, there have been three published studies reporting brain volume differences in chronic ketamine users relative to controls, the first finding lower frontal cortical volume (Liao et al. 2011), the second finding lower grey and white matter volumes in frontal, parietal, occipital and cerebellar cortices (Wang et al. 2013), and the most recent study finding lower parietal and frontal cortex grey matter volumes (Hung et al. 2020). However, these studies used non-drug using controls, and where reported, the control groups had much lower alcohol and tobacco use (Liao et al. 2011), and so the findings may have been due, at least in part, to other substances that the ketamine users may have been taking. Furthermore, none of the studies reported the relationship between brain volume and psychosis-like symptoms in the ketamine users so it is unclear if volumetric differences are linked to the clinical effects of chronic ketamine use.

To address this question, in the current study, we test the hypothesis that chronic ketamine users have reduced subcortical and cortical grey matter volume and increased levels of sub-threshold psychotic symptoms and that the cortical brain volume changes are correlated with the degree of symptom intensity.

## Methods

This study utilises previously unreported volumetric MRI data acquired as part of other published studies from our group (Freeman et al. 2013; Morgan et al. 2014; Stone et al. 2014).

Thirty-six participants were recruited from a database of existing drug users at the clinical psychopharmacology unit, University College London (UCL) and via word of mouth. After giving informed consent, all participants were required to provide a self-report of their drug use history. Exclusion criteria included history of self-reported mental illness requiring psychiatric input, history of head injury, left-handedness and non-fluency in English. Seventeen ketamine users (with ketamine use at least three times per week for the past year) and 19 poly-drug using controls (with no history of regular ketamine use) were recruited through advertising and word of mouth. Participants were required to abstain from psychotropic drugs for at least 24 h before the study. Abstinence from recent drug use was confirmed with urine dipstick analysis. The study was approved by both the Imperial College Research Ethics Committee and the UCL Graduate School ethics committee. All participants were compensated for their time and travel expenses.

### Assessment of sub-threshold psychotic symptoms

All participants were assessed for sub-threshold psychotic symptoms using the Comprehensive Assessment of At-Risk Mental State (CAARMS), a semi-structured assessment tool used to identify individuals at high risk of developing psychosis (Yung et al. 2005), and for schizotypal symptoms using the Schizotypal Personality Questionnaire (SPQ), a self-report scale of schizotypy (Raine 1991).

### Structural MRI acquisition

MRI imaging on a Philips 3-T Intera magnetic resonance system, software release 2.1.3., was then completed. An initial localiser scan was performed followed by acquisition of a whole-brain 3D-MPRAGE (TR = 9.6 ms, TE = 4.5 ms, flip angle 8°, slice thickness = 1.2 mm, 0.94 mm × 0.94 mm in plane resolution, 150 slices).

### Regional brain volume analysis

A region of interest (ROI)-based method of cortical reconstruction and volumetric segmentation was performed using the *Freesurfer* image analysis suite (Freeman et al. 2013), which is documented and freely available for download online (http://surfer.nmr.mgh.harvard.edu/). This processing includes motion correction and averaging of T1-weighted images to remove acquisition artefacts and removal of non-brain tissue using a hybrid watershed/surface deformation procedure followed by automated Talairach transformation to align each MR image to a reference image before segmenting the subcortical white matter and deep grey matter volumetric structures into regions of interest. Intensity normalisation was then performed to improve the signal–noise ratio followed by computer-defined delineation of the cortical grey matter/white matter boundary and grey matter/cerebrospinal fluid border. The cerebral cortex was automatically parcellated according to the Desikan-Killiany regions of interest (Desikan et al. 2006). Cortical grey matter volume, cortical thickness, cortical surface area, subcortical white matter volume and deep grey matter volume ROI measurements were calculated.

### Statistical analysis

Multivariate general linear model (GLM) statistics were performed using *IBM SPSS statistics 22* to determine group-level differences in individual cortical ROI volumes, ROI thickness and surface area measurements, as well as cortical volume (as a whole) and subcortical grey and white matter volume. Total intracranial volume (TIV) was used as a covariate in statistical modelling of volume differences. *Mathworks MATLAB R2014a* was used for false discovery rate (FDR) correction (q < 0.05) of multiple comparisons (Genovese et al., 2002; Glickman et al., 2014). Uncorrected partial correlations were performed in *IBM SPSS statistics 22* to investigate potential associations between sub-threshold psychotic symptoms and level of ketamine use with the volume of the nucleus accumbens, caudate nucleus, cerebral cortex and cerebellar cortex, within ketamine users only.

## Results

### Demographics

Ketamine users did not differ significantly from poly-drug using controls in terms of age, sex or years in education (Table 1). They also did not differ from controls in terms of whether they used alcohol, tobacco or cannabis but were significantly more likely to be users of 3,4-methylenedioxymethamphetamine (MDMA, ‘ecstasy’), cocaine, amphetamine and heroin. Furthermore, they used alcohol, MDMA and cannabis more frequently and had a longer history of use of all substances, with the exclusion of heroin (Table 1). Ketamine users had significantly higher levels of sub-threshold psychotic symptoms, as measured by the CAARMS, and higher levels of schizotypal features on the SPQ than poly-drug using controls (Table 2). Three of the ketamine users reached criteria for psychosis, based on severity and frequency of their experiences as measured using the CAARMS.

**Table 1:** Demographics and drug use in ketamine users and poly-drug using controls

	Poly-drug controls (n = 19)	Ketamine users (n = 17)	Test statistic	p-value
Age (mean (SD))	25.5 (4.9)	28.7 (5.1)	t = 1.9	0.07
Sex (male/female)	13/6	12/5	χ^2^ = 0.02	0.9
Years in education (mean (SD))	16.8 (3.6)	14.6 (3.5)	t = 1.9	0.07
Ketamine use				
Number of regular users	0	17	-	
Amount used (g/session) (mean (SD))	0.08 (0.23)	4 (3.4)	t = 4.66	< 0.001
Frequency (days per month) (mean (SD))	0.004 (0.02)	17.4 (11.1)	t = 6.82	< 0.001
Years of use (mean (SD))	0.1 (0.3)	10.5 (2.7)	t = 16.8	< 0.001
Other drugs used				
Alcohol	19	17	-	1
Tobacco (cigarette smoking)	8	9	χ^2^ = 1.84	0.4
Cannabis	17	17	χ^2^ = 1.9	0.2
MDMA	9	17	χ^2^ = 12.4	< 0.001
Cocaine	9	16	χ^2^ = 12.9	0.002
Amphetamine	5	16	χ^2^ = 20.7	< 0.001
Heroin	0	6	χ^2^ = 11.49	0.003
Drug use (times per month)				
Alcohol (mean (SD))	7.2 (5.3)	13.3 (10.7)	t = 2.131	0.04
Tobacco (mean (SD))	11.7 (12.5)	17.4 (14.6)	t = 1.25	0.2
Cannabis (mean (SD))	3.6 (8.2)	16.7 (13.8)	t = 3.47	0.002
MDMA (mean (SD))	0.2 (0.5)	1.08 (1.3)	t = 2.67	0.01
Cocaine (mean (SD))	0.7 (2.3)	2.17 (3.0)	t = 1.66	0.1
Amphetamine (mean (SD))	0.0 (0)	4.4 (10.0)	t = 1.79	0.09
Heroin (mean ± SD)	0.0 (0)	0.4 (0.9)	t = 1.74	0.1
Drug use (years of use)				
Alcohol (mean (SD))	10.1 (6.1)	14.6 (6.3)	t = 2.16	0.04
Tobacco (mean (SD))	5.6 (7.0)	12.8 (7.4)	t = 2.99	0.005
Cannabis (mean (SD))	3.6 (4.1)	12.9 (6.2)	t = 5.32	< 0.001
MDMA (mean (SD))	1.4 (2.5)	9.4 (5.1)	t = 6.03	< 0.001
Cocaine (mean (SD))	1.6 (2.8)	7.4 (4.1)	t = 4.94	< 0.001
Amphetamine (mean (SD))	0.3 (0.9)	8.1 (6.7)	t = 5.08	< 0.001
Heroin (mean (SD))	0 (0)	1.8 (4.2)	t = 1.83	0.076

**Table 2:** Levels of sub-threshold psychotic symptoms, as measured using the Comprehensive Assessment of At-Risk Mental State (CAARMS), and schizotypal features, as measured using the Schizotypal Personality Questionnaire (SPQ), in ketamine users and poly-drug using controls

	Poly-drug controls (n = 19)	Ketamine users (n = 17)	Test statistic	p-value
CAARMS severity				
Abnormalities of thought content (mean ± SD)	0.4 ± 19	2.5 ± 2	t = 4.2	< 0.001
Abnormal perceptions (mean ± SD)	1.6 ± 1.8	3.0 ± 1.8	t = 2.4	0.02
Abnormalities of speech production (mean ± SD)	1.1 ± 1.5	2.2 ± 1.8	t = 1.9	0.06
CAARMS frequency				
Abnormalities of thought content (mean ± SD)	0.2 ± 0.5	2.6 ± 2.2	t = 4.5	< 0.001
Abnormal perceptions (mean ± SD)	0.7 ± 1.4	2.6 ± 1.8	t = 3.5	0.001
Abnormalities of speech production (mean ± SD)	0.6 ± 1.5	2.8 ± 2.0	t = 3.4	0.002
SPQ				
Total score (mean ± SD)	11.0 ± 7.3	27.6 ± 20.6	t = 3.2	0.003
Cognitive and perceptual (mean ± SD)	2.6 ± 2.8	9.1 ± 8.4	t = 3.1	0.004
Interpersonal (mean ± SD)	4.2 ± 3.2	9.7 ± 9.0	t = 2.5	0.018
Disorganisation (mean ± SD)	3.8 ± 4.8	8.7 ± 5.2	t = 2.8	0.008

### Global and regional brain volume measurements

*Freesurfer*-based analysis of the apparent volumes of gross cerebral structures revealed that ketamine users had statistically significantly smaller volumes of the whole cerebral cortex, cerebellar cortex, nucleus accumbens and caudate nucleus (all FDR p < 0.05; Cohen’s d 0.36–0.75; Table 3). By contrast, the volumes of the pallidum, putamen, corpus callosum, thalamus, cerebellar white matter, amygdala, hippocampus and total cerebral white matter did not differ significantly between the groups. When excluding the 3 ketamine users who reached criteria for psychosis, the difference in total cerebral cortex volume no longer reached statistical significance (FDR p = 0.051), but otherwise the findings were unchanged. To explore the cortical volume changes in more detail, we next carried out a detailed region of interest analysis of cortical regions using the 34 pre-parcellated cortical ROIs available in *Freesurfer*. This revealed that ketamine users had statistically significantly smaller grey matter volumes of the frontal cortex (caudal middle frontal gyrus, superior frontal gyrus and paracentral gyrus), temporal cortex (temporal pole, superior temporal gyrus, middle temporal gyrus and banks of the superior temporal sulcus) and parietal cortex (inferior parietal cortex, supramarginal gyrus and isthmus of cingulate cortex) (FDR p < 0.05; Cohen’s d 0.7–1.31; Table 4). When excluding the 3 ketamine users who reached criteria for psychosis, lower grey matter volumes in caudal middle frontal gyrus, paracentral gyrus, temporal pole, inferior parietal cortex and supramarginal gyrus of ketamine users compared to controls remained statistically significant (FDR p < 0.05). In this post-hoc analysis, the volumes of the remaining ROIs were no longer statistically significant between the groups (all p < 0.1).

**Table 3:** Gross brain structures showing significant (FDR-controlled *p* < 0.05) differences in brain volume between ketamine users and poly-drug using controls

	Poly-drug controls		Ketamine users						
Structure	Mean (cm^3^)	SEM	Mean (cm^3^)	SEM	F-statistic	p-value	q-value	Difference in volume (%)	Cohen’s d
Accumbens	1.29	0.03	1.14	0.03	10.74	< 0.001	< 0.001	− 11.64	0.79
Caudate	8.07	0.19	7.34	0.20	7.31	0.01	0.03	− 9.05	0.54
Cerebellum	131.01	2.26	121.95	2.39	7.52	0.01	0.03	− 6.92	0.56
Cerebral cortex	572.91	8.19	540.95	8.66	7.14	0.01	0.03	− 5.58	0.36

**Table 4:** Cortical regions showing significant (FDR-controlled *p* < 0.05) differences in brain volume between ketamine users and poly-drug using controls

	Poly-drug controls		Ketamine users						
Region of interest	Mean (cm^3^)	SEM	Mean (cm^3^)	SEM	F-statistic	p-value	q-value	Difference in volume (%)	Cohen’s d
Inferior parietal cortex	31.73	0.71	27.67	0.75	15.32	< 0.001	< 0.001	− 12.8	1.31
Temporal pole	5.08	0.11	4.55	0.11	11.52	< 0.001	< 0.001	− 10.51	1.13
Caudal middle frontal gyrus	16.19	0.55	13.78	0.58	9.06	< 0.001	< 0.001	− 14.89	1.01
Supramarginal gyrus	25.43	0.55	23.21	0.58	7.68	0.01	< 0.001	− 8.74	0.92
Paracentral gyrus	8.02	0.17	7.38	0.18	7.11	0.01	< 0.001	− 8.08	0.88
Middle temporal gyrus	26.02	0.56	24.06	0.59	5.81	0.02	< 0.001	− 7.55	0.81
Superior frontal gyrus	52.08	1.06	48.34	1.13	5.80	0.02	< 0.001	− 7.18	0.81
Isthmus of cingulate cortex	5.31	0.14	4.84	0.15	5.34	0.03	< 0.001	− 8.87	0.77
Superior temporal gyrus	27.02	0.62	25.03	0.65	4.89	0.03	< 0.001	− 7.37	0.74
Banks of superior temporal sulcus	6.18	0.22	5.51	0.23	4.46	0.04	< 0.001	− 10.92	0.70

Ketamine users were also found to have a smaller surface area of the temporal pole and inferior parietal cortex (FDR p < 0.05). There were no regional differences in cortical thickness between the groups that survived FDR correction.

### Associations between ketamine use, sub-threshold psychotic symptoms and brain volumes

**Fig. 1:**
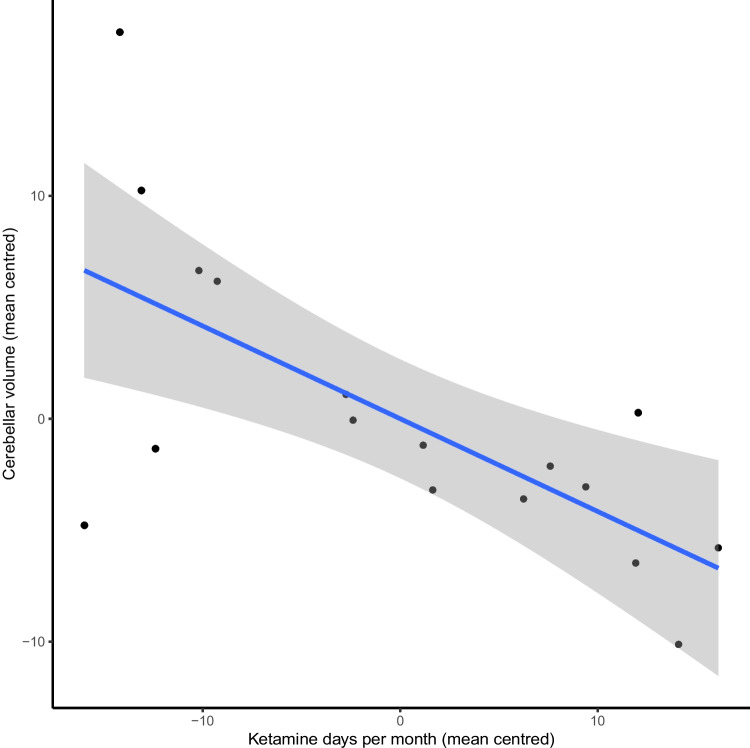
Partial correlation between ketamine use per month and cerebellar volume (corrected for total intracranial volume)

Within the ketamine user group, the frequency of ketamine use inversely correlated with the volumes of the cerebellar cortex (r =  − 0.816; p < 0.001; Fig. 1). This remained significant after correcting for multiple comparisons, including all other drug, tobacco and alcohol use as covariants (r =  − 0.756; p < 0.05). By contrast, there were no statistically significant relationships between ketamine use and the volumes of any other brain ROI, including cortical regions, nor between sub-threshold psychotic symptoms (assessed using CAARMS) or SPQ scores and the volumes of any brain ROI in ketamine users. There was no correlation between brain volumes and level of use for any of the other drugs of abuse in ketamine users or in poly-drug using controls.

## Discussion

In this study, we found that chronic ketamine users had significantly lower grey matter volumes in the frontal, temporal and parietal cortices. These data are consistent with reports of reduced frontal (Liao et al. 2011) and parietal cortex grey matter volume in chronic ketamine users (Hung et al. 2020) as compared to healthy (i.e. non-poly-drug using) controls. They also match closely with the observations of grey matter reductions in the frontal, temporal and parietal cortices in patients with first episode psychosis and schizophrenia, reported by large-scale meta-analyses of such data (Brugger and Howes 2017; Vita et al. 2012). These findings are also in keeping with a recent mega-analysis of structural neuroimaging data in patients with schizophrenia which found global reductions in cortical surface area accompanied by reductions in frontal and temporal cortical thickness (van Erp et al. 2018).

We also found reduced volumes in caudate, nucleus accumbens and cerebellum in chronic ketamine users. One other group reported lesions in cerebellum and striatum in ketamine users (Wang et al. 2013), but these findings have not been reported in other studies. Another group found greater caudate volumes in poly-drug using ketamine users and pure ketamine users compared to non-drug using controls, as measured by MRI (Liang et al. 2020). The reason for this difference may be because we studied poly-drug users rather than non-drug users as controls. Alternatively, the differences may have arisen due to the heavier use of other drugs in the ketamine users in our study. In contrast, a group studying the effect of intermittent ketamine for treatment of major depressive disorder reported increases in grey matter volume in amygdala and hippocampus (Zhou et al. 2020). It is likely that the processes occurring when ketamine is used intermittently for the treatment of depression differ from chronic ketamine use in the setting of dependence or harmful use and may reflect the increases in dendritic spine formation that are hypothesised to underlie the antidepressant effects of ketamine, as supported by data from pre-clinical studies (Li et al. 2010).

It is interesting to note that the cerebellar volume reductions we found appear to occur in patients with schizophrenia (Moberget et al. 2018), but not those with bipolar affective disorder (Laidi et al. 2015), further strengthening the argument that chronic ketamine use may model some of the underlying neuropathology of schizophrenia. The largest meta-analysis of subcortical MRI studies in schizophrenia found reductions in the volume of the hippocampus as well as the amygdala, thalamus and nucleus accumbens (van Erp et al. 2016). This partially overlaps with our findings, but we did not see any reduction in either hippocampus or thalamus volumes in ketamine users in the present study.

As we reported in two previous overlapping studies (Morgan et al. 2014; Stone et al. 2014), we found increased sub-threshold psychotic symptoms in chronic ketamine users. These symptoms did not correlate with grey matter volume reductions. While correlations between grey matter volume and positive psychotic symptoms have previously been reported in patients with schizophrenia (Padmanabhan et al. 2015), the effect size was small (r of approximately 0.15), requiring a sample of over 100 to detect a significant difference with a power of 0.8. Thus, the present study was not of sufficient size to detect such a relationship and so further studies in larger samples are required to address this with sufficient power. The chronic ketamine users also had increased schizotypal personality symptoms as measured with the SPQ. It is not clear whether these features were longstanding or arose because of the regular ketamine use. Such information would require a longitudinal study and remains to be addressed.

One possible difficulty with using chronic ketamine use as a model to understand the underlying neurobiology of schizophrenia is that some of the chronic ketamine users may have had undeclared (or undiagnosed) schizophrenia. We attempted to address this possibility by recruiting patients who did not have any personal history of mental illness. Nonetheless, three of the ketamine users had symptomatology that reached diagnostic criteria for psychosis. This was not particularly surprising, given previous reports of schizophrenia-like symptomatology in chronic ketamine users (Abi-Saab et al. 1998; Javitt 2007; Jentsch and Roth 1999; Krystal et al. 1994; Morgan et al. 2012). To investigate the possibility that the findings were driven by these individuals, we ran post-hoc analyses from which they were excluded. Although there were fewer regions that were significantly different between controls and ketamine users in these analyses, the same overall finding of reduced grey matter volumes in the cerebellar cortex, nucleus accumbens and caudate nucleus and frontal, temporal and parietal cortical regions was seen. This shows that despite the reduced power due to excluding participants, the overall finding of reduced grey matter in chronic ketamine users compared to poly-drug using controls remained.

There are a number of limitations of the present study. (1) As mentioned, this study was relatively small, with low power to detect group differences, although this can also be attributed to the difficulty of recruiting poly-drug using controls and ketamine users. Future studies should aim, if possible, for a larger group size in order to address more subtle group differences; (2) despite our best efforts, the matching of the two groups was not perfect, with the ketamine users trending towards being older and having fewer years of education. Although these differences did not reach statistical significance, it is not clear to what extent this may have affected the results; (3) similarly, the ketamine and poly-drug users were not fully matched for their other drug use. We chose to match with a poly-drug control group to try to take account of the effect of these drugs on the brain to tease out the effect of ketamine alone. However, ketamine users were more likely to also be users of MDMA, cocaine, amphetamine and heroin than the poly-drug users and were also heavier users of alcohol and cannabis, which is a particular challenge for studies of this type. This means that it is not possible to determine whether the lower brain volumes in ketamine users were primarily due to ketamine use or to the use of these other substances. Users of alcohol, amphetamines, cannabis, cocaine and opiates have shown similar reductions in frontal, parietal and temporal cortices, as well as in cerebellum (Batalla et al. 2013; Berman et al. 2008; Mackey and Paulus 2013; Syaifullah et al. 2020; Wollman et al. 2017). Indeed, it is striking how much overlap there is in regional reductions in grey matter volume with all these drugs of abuse, suggesting they may all have similar potential for toxicity to the brain (Mackey et al. 2019). Despite this, we found that the correlation between level of ketamine use and cerebellar volume was still present after correcting for the frequency of other drug use and that other drug use did not correlate with grey matter volume in any of the regions of interest, suggesting that at least some of the differences in brain grey matter between ketamine users and poly-drug using controls were due to ketamine use. Further controlled, reverse-translational studies on the effects of chronic NMDA antagonists in rodents may be of use to confirm (a) specific effects of ketamine and (b) link these to their cellular and molecular correlates post-mortem (Doostdar et al. 2019).

In conclusion, this study shows that individuals with regular heavy ketamine use had lower grey matter volumes in cortical brain regions, cerebellum, nucleus accumbens and caudate nucleus, as indexed using structural MRI, compared to poly-drug using controls. These findings support the hypothesis that chronic ketamine use may lead to similar changes in grey matter that are seen in patients with first episode psychosis and schizophrenia, but caution is required given that other drugs of abuse with non-glutamatergic mechanisms of action lead to similar changes.

## Supplementary Information

Below is the link to the electronic supplementary material.

Supplementary file1 (MSG 85 KB)
